# TREM-1 Protects HIV-1-Infected Macrophages from Apoptosis through Maintenance of Mitochondrial Function

**DOI:** 10.1128/mBio.02638-19

**Published:** 2019-11-12

**Authors:** Grant R. Campbell, Rachel K. To, Stephen A. Spector

**Affiliations:** a Division of Infectious Diseases, Department of Pediatrics, University of California San Diego, La Jolla, California, USA; b Rady Children’s Hospital, San Diego, California, USA; Université du Québec à Trois-Rivières; CIML

**Keywords:** BCL2 family, TREM-1, apoptosis, human immunodeficiency virus, macrophages, mitochondria, mitofusin

## Abstract

Macrophages are a reservoir for latent human immunodeficiency type 1 (HIV) infection and a barrier to HIV eradication. In contrast to CD4^+^ T cells, macrophages are resistant to the cytopathic effects of acute HIV infection. Emerging data suggest a role for TREM1 (triggering receptor expressed on myeloid cells 1) in this resistance to HIV-mediated cytopathogenesis. Here, we show that upon HIV infection, macrophages increase the expression of BCL2, BCLXL, TREM1, mitofusin 1 (MFN1), and MFN2 and the translocation of BCL2L11 (BIM) to the mitochondria and decrease the expression of BCL2-associated agonist of cell death (BAD) and BAX while maintaining a 95% survival rate over 28 days. The HIV proteins Tat and gp120 and the GU-rich single-stranded RNA (ssRNA) (RNA40) from the HIV long terminal repeat region (and a natural Toll-like receptor 8 [TLR8] agonist) induced similar effects. *TREM1* silencing in HIV-infected macrophages led to decreased expression of BCL2, BCLXL, MFN1, and MFN2 and increased expression of BAD and BAX. This correlated with a significant increase in apoptosis mediated by a disruption of the mitochondrial membrane potential (Δψm), leading to the release of cytochrome *c* and caspase 9 cleavage. Exposure of *TREM1*-silenced macrophages to Tat, gp120, or RNA40 similarly resulted in the disruption of Δψm, cytochrome *c* release, caspase 9 cleavage, and apoptosis. Thus, our findings identify a mechanism whereby HIV promotes macrophage survival through TREM1-dependent upregulation of BCL2 family proteins and mitofusins that inhibits BCL2L11-mediated disruption of Δψm and subsequent apoptosis. These findings indicate that TREM1 can be a useful target for elimination of the HIV reservoir in macrophages.

## Abstract (precis)

The major challenge to human immunodeficiency virus (HIV) treatment is the development of strategies that lead to viral eradication. A roadblock to accomplishing this goal is the lack of an approach that would safely eliminate HIV from all resting/latent reservoirs, including macrophages. Macrophages are a key part of the innate immune system and are responsible for recognizing invading microbes and sending appropriate signals to other immune cells. Here, we found that HIV induces the upregulation of the protein TREM1 (triggering receptor expressed on myeloid cells 1), which signals an increase in the expression of antiapoptotic proteins, thus promoting survival of HIV-infected macrophages.

## INTRODUCTION

The major obstacle to eradicating human immunodeficiency type 1 (HIV-1) is the establishment of long-lived reservoirs of virus. Although latently infected CD4^+^ T cells represent the predominant viral reservoir in infected persons on suppressive antiretroviral therapy (ART), other cell types, including macrophages, serve as important sites of HIV persistence (1–3). Latently HIV-infected perivascular macrophages contain replication-competent proviral HIV DNA, support persistent permissive HIV infection in the absence of CD4^+^ T cells (4, 5), and contain lower intracellular concentrations of antiretrovirals than CD4^+^ T cells, resulting in ongoing HIV replication (6). Latently infected macrophages also harbor and transmit replication-competent virus to nearby CD4^+^ T cells through virologic synapses that are resistant to antibody-mediated neutralization (7). Additionally, they survive for months to years and are resistant both to the cytopathic effects of HIV infection and to CD8^+^ T cell-mediated killing (8–10).

Notwithstanding recent advances defining cell death signaling pathways, much is unknown about the mechanism(s) by which macrophages escape apoptosis during HIV infection. Despite this, studies suggest that HIV signals through multiple pathways to reprogram the transcriptome and proteome of human macrophages to render them resistant to HIV-mediated apoptosis. HIV Nef induces the phosphorylation and inactivation of proapoptotic BCL2-associated agonist of cell death (BAD) (11), and HIV Tat and gp120 induce the transcription of apoptosis regulatory proteins, including colony-stimulating factor 1 (CSF1), caspase 8 (CASP8) and Fas associated via death domain (FADD)-like apoptosis regulator (CFLAR), BCL2 family members BCL2 and BCLXL, and inhibitor of apoptosis proteins (IAP) X-linked inhibitor of apoptosis (XIAP), baculoviral IAP repeat-containing 2 (BIRC2), and BIRC3 (12–14). Additionally, acute exposure to HIV and to HIV gp41 peptides upregulates triggering receptor expressed on myeloid cells 1 (*TREM1*) in peripheral blood mononuclear cells (PBMC) and macrophages (15–17).

TREM1 is a 30-kDa glycoprotein expressed on macrophages that belongs to the immunoglobulin variable (IgV) domain superfamily of proteins and consists of a positive-charged transmembrane domain and a short cytoplasmic domain that lacks any signaling motif. Thus, TREM1 requires TYRO protein tyrosine kinase binding protein (TYROBP) for signaling (18, 19). Although the precise ligands for TREM1 are unknown, CD177, peptidoglycan recognition protein 1 (PGLYRP1), high-mobility group box 1 protein (HMGB1), and some damage-associated molecular patterns (DAMPs) and pathogen-associated molecular patterns (PAMPs) activate TREM1 signaling (20–24). TREM1 ligation leads to an increase in reactive oxygen species and the secretion of proinflammatory cytokines through the activation of NF-κB. Additionally, TREM1 ligation synergistically amplifies Toll-like receptor (TLR)-mediated and Nod-like receptor (NLR)-mediated proinflammatory responses (19). TREM1 is also an antiapoptotic molecule that prolongs macrophage survival. Specific ligation or overexpression of TREM1 induces BCL2 expression and the subsequent depletion of caspase 3, preventing the cleavage of PARP1 (25). In addition, both ligation and overexpression of TREM1 lead to an increase in mitofusin expression levels, suggesting that TREM1 contributes to the maintenance of mitochondrial integrity, thus favoring cell survival (25). The involvement of and mechanisms by which TREM1 may alter macrophage survival during HIV infection are unknown. Therefore, we investigated whether TREM1 affects cell survival during HIV infection of human primary macrophages.

## RESULTS

### HIV infection of macrophages results in persistent infection and prolongs cell survival.

**FIG 1:**
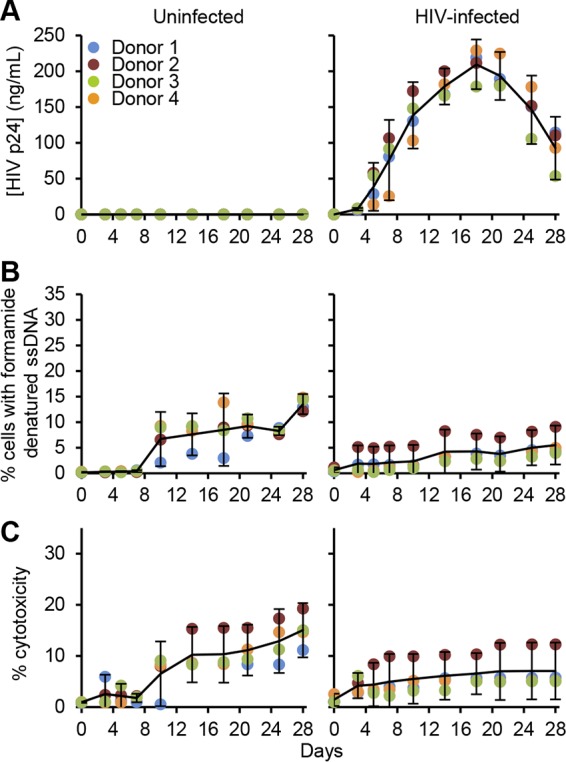
Macrophages were left uninfected (left) or infected with purified HIV (right) at an MOI of 0.002 to allow slow progressive infection of cells. (A) ELISA was performed for supernatant HIV p24 antigen over 28 days, *n* = 4. (B) Cells were fixed and permeabilized, and the percentage of cells with apoptotic ssDNA damage was quantified by ELISA, *n* = 4. (C) Aliquots of supernatants were spectrophotometrically tested for LDH using the LDH^PLUS^ assay, *n* = 4.

We designed our initial experiments to determine whether HIV infection results in increased apoptosis of infected macrophages. We infected macrophages with HIV for 28 days, during which time we assessed HIV replication kinetics and apoptosis. HIV p24 antigen levels increased in culture supernatants between day 3 and day 18 followed by a decline after 18 days (Fig. 1A). Uninfected macrophages showed low levels of apoptosis, with an observed maximum of 13.5% of cells displaying formamide-sensitive single-stranded DNA (ssDNA; a specific indicator of apoptosis [26]) by day 28 (Fig. 1B). HIV infection of macrophages was associated with a decrease in cell death, reaching a high of just 5.5% at day 28 (±0.3% standard error of the mean [SEM]; *P* = 0.017) (Fig. 1B). Culture supernatants assessed for lactate dehydrogenase (LDH) release demonstrated the same profile (Fig. 1C). Collectively, these results suggest that HIV infection prolongs survival of macrophages.

### HIV influences pro- and antiapoptotic protein expression in macrophages.

**FIG 2:**
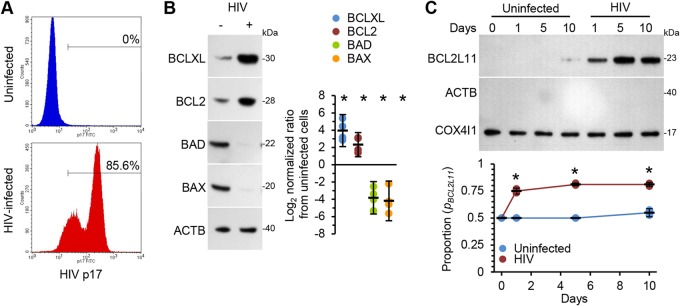
Macrophages were left uninfected or infected with HIV for 10 days. (A) At day 10 postinfection, cells were harvested, stained for HIV p17 expression, and analyzed by flow cytometry. Representative histogram plots are shown, *n* = 3. (B) (Left) Representative Western blots of BCLXL, BCL2, BAD, and BAX with antibodies against BCLXL, BCL2, BAD, BAX, and ACTB. (Right) Densitometric analysis of blots, *n* = 4. (C) Mitochondria were extracted over time. (Top) Representative Western blots of BCL2L11 with antibodies against BCL2L11, ACTB, and COX4I1. ACTB was used a control for mitochondrion fraction purity. (Bottom) Densitometric analysis of blots, *n* = 4.

To determine why there is an absence of apoptosis in HIV-infected macrophages, we analyzed the induction of proapoptotic (BAD and BAX) and antiapoptotic (BCLXL and BCL2) proteins in HIV-infected macrophages (Fig. 2A). At day 10 postinfection, the expression levels of two antiapoptotic factors, BCL2 and BCLXL, were increased (*P* < 0.01) whereas those of the proapoptotic proteins BAX and BAD were decreased (*P* < 0.01; Fig. 2B). Next, we determined if the HIV antigens gp120, Tat, and RNA40 (a 20-mer synthetic single-stranded RNA oligoribonucleotide derived from the HIV long-terminal repeat and TLR8 ligand) influenced the expression of these pro- and antiapoptotic proteins in macrophages. Both gp120 and Tat increased the expression levels of BCL2 and BCLXL in a dose-dependent manner while simultaneously decreasing those of both BAD and BAX (see Fig. S1 in the supplemental material). In contrast, while RNA40 also dose dependently increased the expression of both BCL2 and BCLXL, it also increased the expression of BAD while having no effect on BAX expression levels (Fig. S1).

10.1128/mBio.02638-19.1 FIG S1 Uninfected macrophages were treated with vehicle, Tat, gp120, RNA41 (R41), or RNA40 for 24 h. (Top) Representative Western blots of BCLXL, BCL2, BAD, and BAX with antibodies against BCLXL, BCL2, BAD, BAX, and ACTB are shown. (Bottom) Densitometric analysis of blots, *n* = 4. Download FIG S1, TIF file, 2.9 MB. Copyright © 2019 Campbell et al. 2019 Campbell et al. This content is distributed under the terms of the Creative Commons Attribution 4.0 International license.

A key event in apoptosis is the translocation of BCL2L11 from the cytoskeleton to the mitochondrial membrane (27). In healthy cells, BCL2L11 is inactivated by its interaction with dynein light chain LC8-type 1 (DYNLL1), which recruits it to the microtubule-based dynein motor complex (28). During HIV infection of CD4^+^ T cells, HIV Tat binds tubulin through a four-amino-acid subdomain of its conserved core region, altering microtubule dynamics (29, 30) and leading to the mitogen-activated protein kinase 8 (MAPK8)-mediated phosphorylation of BCL2L11 and dissociation from DYNLL1 and the dynein motor complex. Phosphorylated BCL2L11 then translocates to the mitochondrial membrane, where it directly activates the BAX/BAK-dependent mitochondrial pathway of apoptosis and cell death (27, 31, 32). Therefore, we examined whether HIV infection also induces the translocation of BCL2L11 to mitochondria in macrophages despite the absence of HIV-mediated cytopathogenesis. Using cell fractionation, we determined that BCL2L11 translocates to mitochondria, suggesting that HIV is inhibiting the mitochondrial pathway of apoptosis (Fig. 2C).

### HIV infection increases the expression of TREM1 in macrophages.

**FIG 3:**
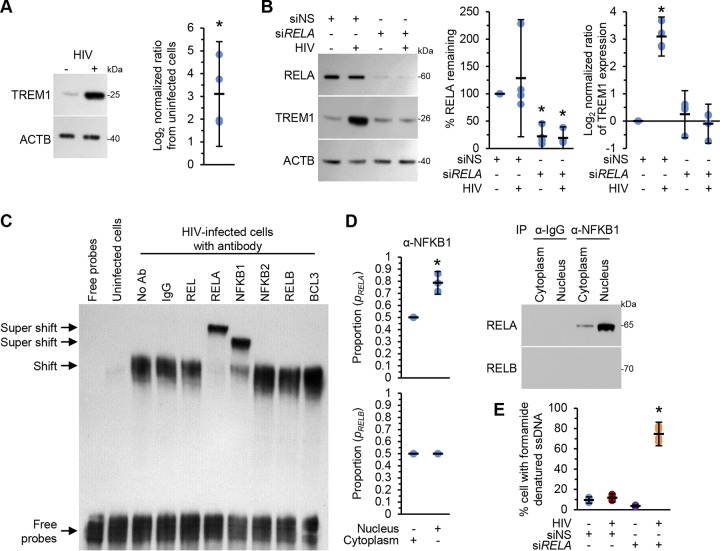
Macrophages were left uninfected or infected with HIV for 10 days. (A) Cells were harvested and lysed. (Left) Representative Western blots of TREM1 with antibodies against TREM1 and ACTB. (Right) Densitometric analysis of blots, *n* = 4. (B) Cells were transfected with *RELA* siRNA (si*RELA*) or scrambled siRNA (siNS). (Left) Representative Western blots of RELA, TREM1, and ACTB. (Right) Densitometric analysis of blots, *n* = 4. (C) Nuclear extracts were prepared and assayed by supershift EMSA using a *TREM1* probe and antibodies specific for human REL, RELA, NFKB1, NFKB2, RELB, and BCL3. A representative blot is shown, *n* = 4. Ab, antibody. (D) Cytoplasmic and nuclear fractions were immunoprecipitated (IP) with anti-NFKB1 and were then subjected to Western blotting using anti-RELA or anti-RELB antibodies. (Right) Representative Western blots. (Left) Densitometric analysis of blots, *n* = 4. (E) Transfected cells from the experiment whose results are presented in panel B were assessed for apoptotic ssDNA damage by ELISA, *n* = 4.

As HIV infection resulted in increased expression of BCL2 and as overexpression of TREM1 can induce the expression of BCL2 (25), we examined whether macrophages productively infected with HIV express higher levels of TREM1. At 10 days after infection, HIV-infected macrophages displayed increased levels of TREM1 (*P* = 0.02; Fig. 3A). We then investigated the mechanism by which HIV induces *TREM1* expression. As NF-κB transcriptionally regulates *TREM1* in macrophages in response to LPS (33), we utilized RNA interference (RNAi) for the NF-κB subunit *RELA* to investigate whether NF-κB regulates *TREM1* expression in response to HIV infection. *RELA* silencing resulted in the almost complete ablation of TREM1 expression in response to HIV (Fig. 3B), suggesting that NF-κB regulates *TREM1* expression in HIV-infected macrophages. To confirm the sequence-specific interaction of RELA with the *TREM1* promoter, we analyzed the promoter region of *TREM1* using Genomatix (version 3.11; Intrexon) and found consensus binding sites for NF-κB. We performed an electrophoretic mobility shift assay using a biotinylated probe that encompassed the predicted binding site. This probe showed evidence of strong binding to nuclear proteins and was supershifted by antibodies against RELA and NFKB1 but not by antibodies against REL, NFKB2, RELB, or BCL3 (Fig. 3C). To investigate whether HIV triggers the classical NF-κB signaling pathway, we analyzed the ability of RELA to physically interact with NFKB1 p50 and to translocate to the nucleus using coimmunoprecipitation. NFKB1 p50/RELA complexes were detected in both the cytoplasm and the nucleus, whereas no NFKB1 p50/RELB complexes were detected (Fig. 3D). Interestingly, although uninfected cells transfected with si*RELA* showed apoptosis levels similar to the levels seen with those transfected with control RNAi, *RELA*-silenced HIV-infected macrophages showed an increased number of apoptotic cells (Fig. 3E; *P* < 0.001).

**FIG 4:**
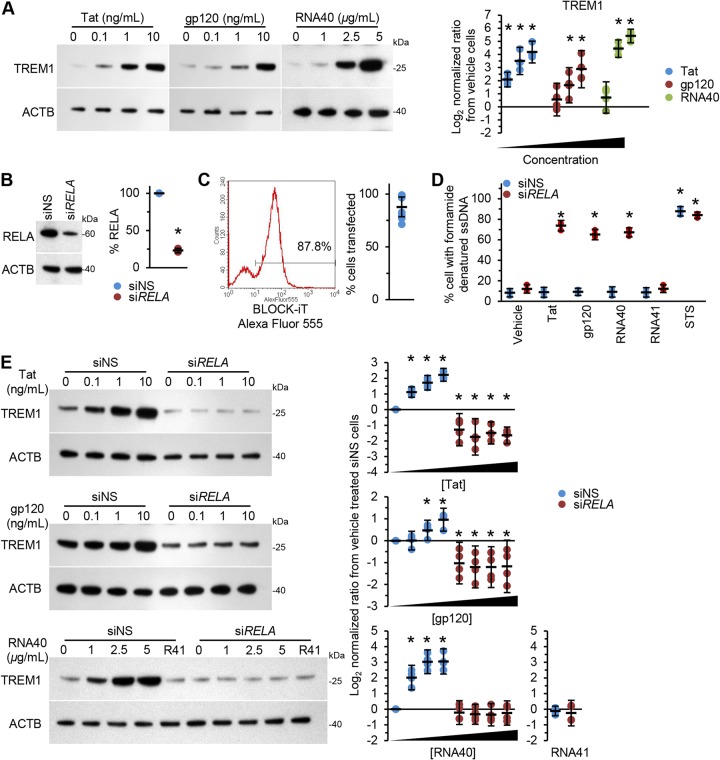
(A) Uninfected macrophages were treated with vehicle, Tat, gp120, or RNA40 for 24 h. (Left) Representative Western blots of TREM1 with antibodies against TREM1 and ACTB. (Right) Densitometric analysis of blots, *n* = 4. (B) Uninfected macrophages were transfected with si*RELA* or siNS for 48 h. (Left) Representative Western blots of RELA with antibodies against RELA and ACTB. (Right) Densitometric analysis of blots, *n* = 4. (C) The transfection efficiency of Lipofectamine RNAiMAX in macrophages was assessed using BLOCK-iT Alexa Fluor red fluorescent control. Cells were harvested 24 h posttransfection and analyzed by flow cytometry. (Left) A representative histogram is shown. (Right) Percentages of cells positive for BLOCK-iT Alexa Fluor red, *n* = 8. (D) Uninfected macrophages were transfected with si*RELA* or siNS for 48 h and then treated with vehicle, Tat, gp120, RNA40, RNA41 (R41), or staurosporine (STS) for 24 h. Apoptotic ssDNA damage was quantified by ELISA, *n* = 4. (E) Uninfected macrophages were transfected with si*RELA* or siNS for 48 h and then treated with vehicle, Tat, gp120, RNA40, or RNA41 (R41) for 24 h. (Left) Representative Western blots of TREM1 with antibodies against TREM1 and ACTB. (Right) Densitometric analysis of blots, *n* = 4.

We next investigated whether HIV gp120, Tat, and RNA40 induce TREM1 expression in human macrophages. All three antigens induced a dose-dependent increase in TREM1 expression; HIV Tat and gp120 induced 18-fold (±3.9 SEM; *P* = 0.0005) and 7.5-fold (±2.9 SEM; *P* = 0.0075) increases, respectively, at 10 ng ml^−1^, and HIV RNA40 induced a 42-fold increase (±4.6 SEM; *P* < 0.0001) at 5 μg ml^−1^ (Fig. 4A). Similarly to HIV-infected macrophages, *RELA* silencing of uninfected macrophages (Fig. 4B and C) significantly increased cell death in response to the presence of HIV Tat, gp120, or RNA40 (Fig. 4D), while it also attenuated TREM1 expression (Fig. 4E). Electrophoretic mobility shift assay (EMSA) demonstrated that HIV Tat, gp120, and RNA40 rapidly induced NF-κB DNA binding, with supershift analysis demonstrating that the NF-κB complex consisted primarily of NFKB1 and RELA subunits (Fig. S2A). We then investigated whether these antigens trigger the classical NF-κB signaling pathway. HIV Tat, gp120, and RNA40 all triggered the rapid processing of NFKB1 p105 to p50, the dimerization of NFKB1 p50 with RELA, and the subsequent nuclear translocation (Fig. S2B and C).

10.1128/mBio.02638-19.2 FIG S2 Uninfected macrophages were treated with vehicle, 10 ng ml^−1^ Tat, 10 ng ml^−1^ gp120, or 5 μg ml^−1^ RNA40. (A) Nuclear extracts were prepared after 24 h and assayed by supershift EMSA using the *TREM1* probe and antibodies specific for human REL, RELA, NFKB1, NFKB2, RELB, and BCL3. Representative blot is shown, *n* = 2. (B) At the time points indicated, cells were harvested and nuclear and cytoplasmic extracts prepared. (Left) Representative Western blots of RELA and NFKB1 with antibodies against RELA, NFKB1, and ACTB. (Right) Densitometric analysis of blots, *n* = 4. (C) After 24 h, nuclear extracts were prepared, immunoprecipitated (IP) with anti-NFKB1, and then subjected to Western blotting using anti-RELA or anti-RELB antibodies. (Left) Representative Western blots. (Right) Densitometric analysis of blots, *n* = 4. Download FIG S2, TIF file, 2.5 MB. Copyright © 2019 Campbell et al. 2019 Campbell et al. This content is distributed under the terms of the Creative Commons Attribution 4.0 International license.

### TREM1 is required for HIV-infected macrophage survival.

**FIG 5:**
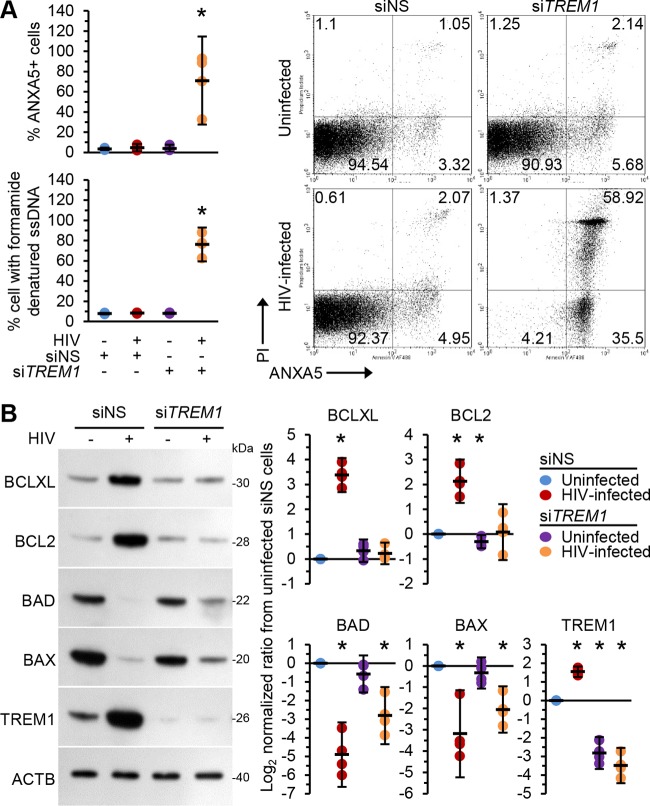
Uninfected macrophages and macrophages infected with HIV for 10 days were transfected with si*TREM1* or siNS for 48 h. (A) Cells were assessed for cell death. (Top left) Cells were harvested and stained with annexin V (ANXA5) and PI. The frequency of ANXA5-positive or ANXA5/PI-positive labeling is illustrated as a plot. (Right) Representative dot plots representing cells from a single donor are shown. (Bottom left) Quantification of the number of cells with apoptotic ssDNA damage by the use of ssDNA ELISA, *n* = 4. (B) Cells were lysed and subjected to Western blotting. (Left) Representative Western blots of BCLXL, BCL2, BAD, BAX, and TREM1 with antibodies against BCLXL, BCL2, BAD, BAX, TREM1, and ACTB. (Right) Densitometric analysis of blots, *n* = 4.

To address the role of TREM1 in macrophage survival during HIV infection, we used *TREM1* small interfering RNA (siRNA). We reasoned that if TREM1 is involved in promoting cell survival, then silencing *TREM1* should increase cell death during HIV infection. Macrophages infected with HIV for 10 days were transfected with control scrambled siRNA (siNS) or *TREM1* siRNA (si*TREM1*) for 48 h and then either assessed for apoptotic ssDNA damage or stained with annexin A5 (ANXA5) and propidium iodide (PI) (Fig. 5A). Uninfected cells transfected with si*TREM1* showed apoptosis levels similar to those transfected with siNS. In contrast, *TREM1* siRNA-transfected HIV-infected macrophages showed an increased number of apoptotic cells (83.9% ± 6%) compared with HIV-infected macrophages transfected with siNS (4.2 ± 1.4%; *P* = 0.02). Because *TREM1* silencing induced apoptosis in productively infected cells, we posited that *TREM1* silencing altered the balance of anti- and proapoptotic proteins. Indeed, *TREM1* silencing resulted in a significant reduction in HIV-induced BCLXL and BCL2 expression (*P* < 0.0003) while having a modest impact on BAD and BAX expression (*P* > 0.06; Fig. 5B).

**FIG 6:**
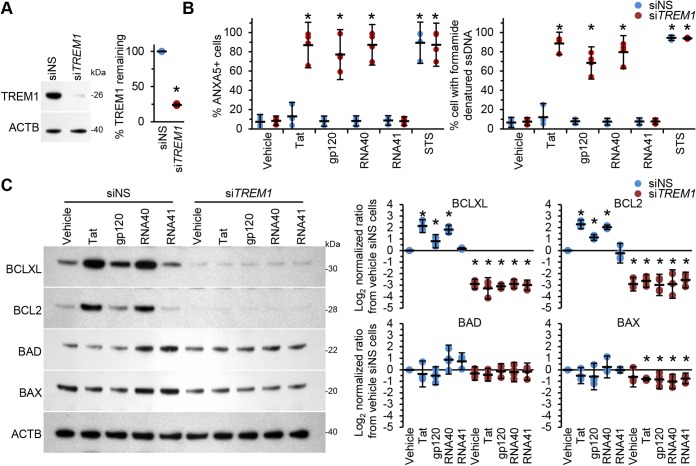
Uninfected macrophages were transfected with si*TREM1* or siNS for 48 h and then treated with vehicle, 10 ng ml^−1^ Tat, 10 ng ml^−1^ gp120, 5 μg ml^−1^ RNA40, or 5 μg ml^−1^ RNA41 for 24 h. (A) Cells were assessed for TREM1 expression. (Left) Representative Western blots of TREM1 with antibodies against TREM1 and ACTB. (Right) Densitometric analysis of blots, *n* = 4. (B) Cells were assessed for cell death. (Left) Cells were harvested and stained with annexin V (ANXA5) and PI. The frequency of ANXA5-positive or ANXA5/PI-positive labeling is illustrated as a plot. (Right) Quantification of the number of cells with apoptotic ssDNA using an ssDNA ELISA, *n* = 4. (C) Cells were lysed and subjected to Western blotting. (Left) Representative Western blots of BCLXL, BCL2, BAD, and BAX with antibodies against BCLXL, BCL2, BAD, BAX, and ACTB. (Right) Densitometric analysis of blots, *n* = 4.

Next, we analyzed the effect of HIV antigen-induced apoptosis on *TREM1*-silenced cells (Fig. 6A) after exposure of the cells to HIV antigens for 24 h. Importantly, exposure of siNS-transfected cells to HIV Tat, gp120, or RNA40 did not result in increased apoptosis (*P* > 0.4; Fig. 6B). In contrast, and similarly to HIV infection, *TREM1*-silenced macrophages treated with HIV antigens displayed an enhanced number of apoptotic cells compared with vehicle-treated cells (Fig. 6B). *TREM1*-silenced cells exposed to Tat exhibited the highest proportion of apoptotic cells (mean, 92.4 ± 0.4%; *P* < 0.0001) closely followed by RNA40 (mean, 84.7% ± 1.3%; *P* < 0.0001) and gp120 (mean, 73.5% ± 2.2%; *P* < 0.0001). Importantly, staurosporine exposure similarly induced apoptosis in both siNS-transfected and si*TREM1*-transfected cells (mean, 94.5% ± 1.4% versus 94.0% ± 0.4%; *P* = 0.4) (Fig. 6B). We then evaluated the effect of *TREM1* silencing on HIV antigen-induced expression of antiapoptotic and proapoptotic proteins. *TREM1* silencing decreased BCLXL and BCL2 expression in uninfected macrophages by more than 86% (*P* < 0.001) while having no noticeable effect on the expression of either BAX or BAD (Fig. 6C). Of note, neither HIV Tat nor gp120 nor RNA40 had a significant effect on the expression of BCLXL, BCL2, BAX, or BAD compared to the vehicle in these *TREM1*-silenced cells. Collectively, these data suggest that induction of TREM1 by HIV inhibits apoptosis and prolongs survival of HIV-infected macrophages.

**FIG 7:**
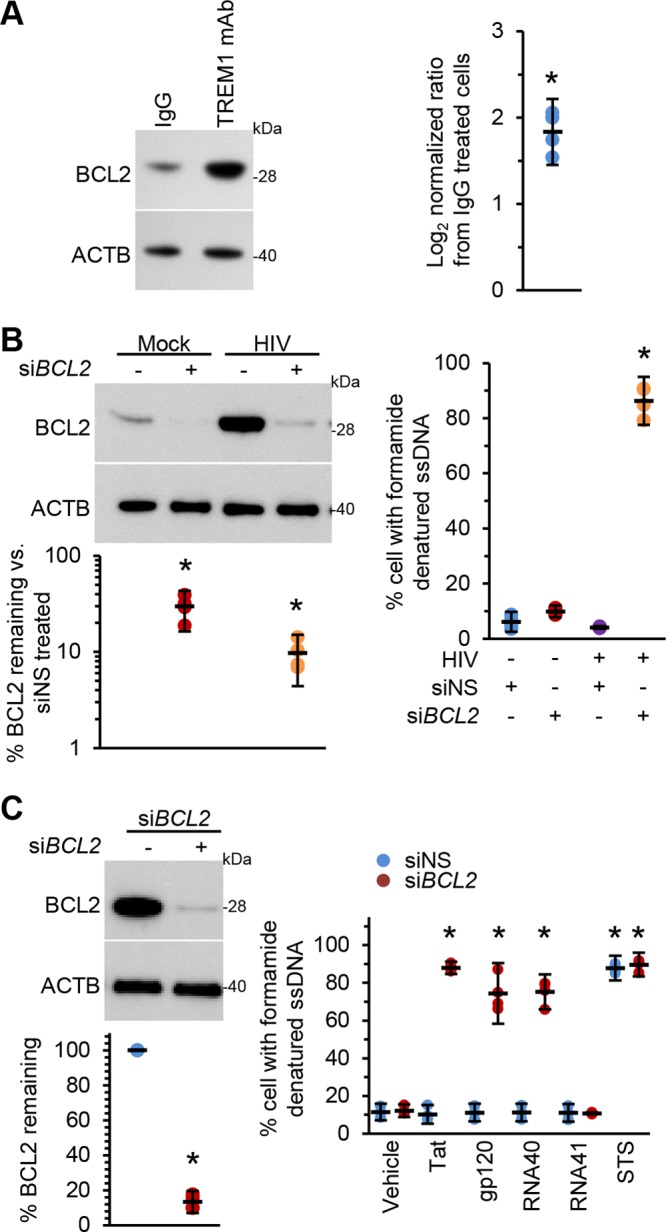
(A) Uninfected macrophages were exposed to 10 μg ml^−1^ TREM1 monoclonal antibody (mAb) for 16 h. Cells were then lysed and assessed by Western blotting. (Left) Representative Western blots of BCL2 with antibodies against BCL2 and ACTB. (Right) Densitometric analysis of blots, *n* = 4. (B) Uninfected macrophages and macrophages infected for 10 days with HIV were transfected with si*BCL2* or siNS for 48 h. (Left top) Cells were lysed and subjected to Western blotting with antibodies against BCL2 and ACTB. (Left bottom) Densitometric analysis of blots. (Right) quantification of the number of cells with apoptotic ssDNA by the use of ssDNA ELISA, *n* = 4. (C) Uninfected macrophages were transfected with si*BCL2* or siNS for 48 h. (Left top) Cells were lysed and subjected to Western blotting with antibodies against BCL2 and ACTB. (Left bottom) Densitometric analysis of blots. (Right) quantification of the number of cells with apoptotic ssDNA by the use of ssDNA ELISA, *n* = 4.

Next, we sought to define the expression of BCL2 in relation to specific ligation of TREM1 by the use of monoclonal antibodies (mTREM1). BCL2 expression was significantly increased in macrophages in response to mTREM1 compared with the isotype control antibody (*P* = 0.0006; Fig. 7A). We then sought to define the relationship between BCL2 expression and cell survival during HIV infection of macrophages using RNAi for BCL2. BCL2 silencing of HIV-infected macrophages led to an increased number of apoptotic cells (86.3% ± 5.4%; *P* = 0.0001) (Fig. 7B). Similarly, BCL2 silencing also significantly increased uninfected macrophage cell death in response to exposure to HIV Tat, gp120, or RNA40 (*P* < 0.001; Fig. 7C).

### *TREM1* silencing leads to the induction of apoptosis through the intrinsic pathway.

**FIG 8:**
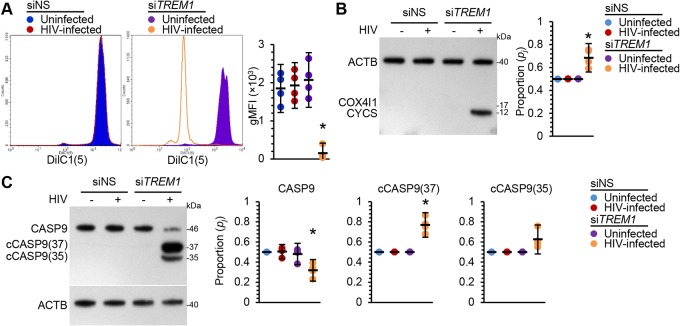
Uninfected macrophages and macrophages infected for 10 days with HIV were transfected with si*TREM1* or siNS for 48 h. Silencing was performed as described for the experiment whose results are shown in Fig. 5B. (A) Cells were stained with DiIC1(5) and then harvested and analyzed by flow cytometry. (Left) Representative histograms of DiIC1(5). (Right) Geometric mean fluorescence intensity illustrated as a plot, *n* = 4. (B) Cells were harvested, fractionated for cytoplasmic content, and assessed by Western blotting. (Left) Representative Western blots of CYCS and COX4I1 with antibodies against CYCS, COX4I1, and ACTB. (Right) Densitometric analysis of blots, *n* = 4. (C) Cells were harvested and assessed by Western blotting. (Left) Representative Western blots of CASP9 and cleaved CASP9 [cCASP9(37) and cCASP9(35)] with antibodies against CASP9 and ACTB. (Right) Densitometric analysis of blots, *n* = 4.

The interactions between anti- and proapoptotic proteins exist in a delicate balance at the mitochondrial membrane that determines cell fate, with mitochondrial fragmentation a central mechanism of apoptosis. Loss of balance between pro- and antiapoptotic BCL2 proteins leads to outer membrane permeabilization of mitochondria, calcium influx, and mitochondrial fragmentation, leading to the subsequent activation of effector caspases. In contrast, a fused mitochondrial phenotype protects cells from programmed cell death. Therefore, we examined the effect of *TREM1* silencing on the mitochondrial membrane potential (Δψm) using DiIC1(5) (1,1′,3,3,3′,3′-hexamethylindodicarbo-cyanine iodide). Following *TREM1* silencing, there was no difference in Δψm levels between siNS-transfected HIV-infected and uninfected macrophages, indicating that mitochondria are still functional during infection. Similarly, we observed no difference in Δψm levels between uninfected cells transfected with si*TREM1* and those transfected with siNS. In contrast, we observed a marked decrease in DiIC1(5) staining intensity in *TREM1*-silenced HIV-infected macrophages (Fig. 8A). These data suggest that *TREM1* silencing results in the depolarization of the Δψm in HIV-infected macrophages but not in uninfected macrophages.

A key event in apoptosis is the formation of the transition pore in the mitochondrial membrane and the release of mitochondrial factors such as cytochrome *c* (CYCS) into the cytoplasm. To determine whether *TREM1* silencing in HIV-infected macrophages leads to the mitochondrial release of CYCS into the cytoplasm, we performed cell fractionation. Our data indicate that mitochondria retain CYCS in siNS-transfected HIV-infected and uninfected cells (Fig. 8B). In contrast, *TREM1* silencing induces the release of CYCS into the cytoplasm in HIV-infected macrophages but not in uninfected macrophages (*P* = 0.018; Fig. 8B). In the presence of ATP/dATP, CYCS released into the cytosol binds to and triggers the oligomerization of cytosolic apoptotic protease activating factor 1 (APAF1). The resultant complex recruits and activates multiple copies of the apical caspase in the mitochondrial pathway of apoptosis, caspase 9 (CASP9). *TREM1* silencing resulted in the cleavage and activation of CASP9 in HIV-infected macrophages but had no effect on CASP9 activation in uninfected macrophages (Fig. 8C).

We next assessed the effect of HIV Tat, gp120, and RNA40 on Δψm, CYCS release, and subsequent CASP9 activation in uninfected macrophages. There was no significant difference in Δψm after Tat, gp120, and RNA40 exposure in siNS-transfected macrophages, indicating that Tat, gp120, and RNA40 have little effect on Δψm in the presence of TREM1 (Fig. S3A). In contrast, we observed a marked decrease in DiIC1(5) staining intensity in *TREM1*-silenced macrophages after Tat, gp120, and RNA40 treatment, suggesting that TREM1 is essential for maintaining Δψm after exposure to HIV antigens. Similarly, treatment of siNS-transfected macrophages with HIV Tat, gp120, or RNA40 did not induce the release of CYCS into the cytosol or induce CASP9 cleavage (Fig. S3B). In contrast, HIV Tat, gp120, and RNA40 exposure of *TREM1*-silenced cells resulted in CYCS release from the mitochondria and subsequent CASP9 cleavage. Together, these results indicate that TREM1 is essential for macrophage survival of acute HIV infection.

10.1128/mBio.02638-19.3 FIG S3 Uninfected macrophages were transfected with si*TREM1* or siNS for 48 h. Cells were then treated with vehicle, 10 ng ml^−1^ Tat, 10 ng ml^−1^ gp120, 5 μg ml^−1^ RNA40, 5 μg ml^−1^ RNA41, or 1 μM staurosporine (STS) for 24 h. Silencing was performed as described for Fig. 6A. (A) Cells were stained with DiIC1(5) and were then harvested and analyzed by flow cytometry. Representative histograms of DiIC1(5) are shown, *n* = 4. (B) Cells were harvested and lysed or fractionated for cytoplasmic content and then assessed by Western blotting. (Left) Representative Western blots of CYCS, COX4I1, CASP9, and cleaved CASP9 with antibodies against CYCS, COX4I1, CASP9, and ACTB. (Right) Densitometric analysis of blots, *n* = 4. Download FIG S3, TIF file, 2.9 MB. Copyright © 2019 Campbell et al. 2019 Campbell et al. This content is distributed under the terms of the Creative Commons Attribution 4.0 International license.

### TREM1 enhances mitochondrial integrity by inducing mitofusin expression.

**FIG 9:**
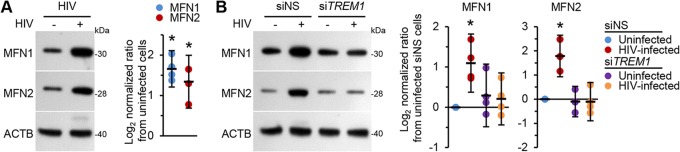
(A) Macrophages were left uninfected or infected with HIV for 10 days. (Left) Representative Western blots of MFN1 and MFN2 with antibodies against MFN1, MFN2, and ACTB. (Right) Densitometric analysis of blots, *n* = 4. (B) Uninfected macrophages and macrophages infected for 10 days with HIV were transfected with si*TREM1* or siNS for 48 h. (Left) Representative Western blots of MFN1 and MFN2 with antibodies against MFN1, MFN2, and ACTB. (Right) Densitometric analysis of blots. Silencing was performed as described for the experiment whose results are shown in Fig. 5B, *n* = 4.

At the molecular level, mitochondrial fusion is a two-step process requiring the coordinated fusion of outer and inner mitochondrial membranes by separable sequential events. In humans, this process depends on distinct mitochondrial sublocalization of three fusogenic proteins: mitofusins 1 and 2 (MFN1 and MFN2) (embedded within the outer mitochondrial membrane) and optic atrophy 1 (located at the inner mitochondrial membrane). In addition to the well-characterized role of the profission dynamin-1-like protein in mediating mitochondrial fission in cells undergoing cell death, inhibition of mitochondrial fusion is also an established consequence of apoptosis induction and occurs around the time of BAX activation. During oxygen glucose deprivation, MAPK1/2 phosphorylates MFN1, inhibiting its profusion function, and triggers its association with and oligomerization of BAK, facilitating the subsequent release of CYCS from mitochondria through BCL2L11 (34). Similarly, in response to cellular stresses, MAPK8 phosphorylates MFN2, leading to its ubiquitin-dependent proteasomal degradation and subsequently to mitochondrial fragmentation and enhanced apoptotic cell death (35). Therefore, we assessed mitofusin expression in HIV-infected macrophages. We found that at day 10 postinfection, the expression levels of both MFN1 and MFN2 were increased (*P* < 0.007; Fig. 9A). We next assessed the effect of HIV Tat, gp120, and RNA40 on mitofusin expression. All three antigens induced dose-dependent increases in both MFN1 and MFN2 (Fig. S4A).

10.1128/mBio.02638-19.4 FIG S4 Uninfected macrophages were transfected with si*TREM1* or siNS for 48 h. (A) Cells were treated with vehicle, Tat, gp120, RNA40, or 5 μg ml^−1^ RNA41 (R41) for 24 h. (Top) Representative Western blots of MFN1 and MFN2 with antibodies against MFN1, MFN2, and ACTB. (Bottom) Densitometric analysis of blots, *n* = 4. (B) Cells were treated with vehicle, 10 ng ml^−1^ Tat, 10 ng ml^−1^ gp120, 5 μg ml^−1^ RNA40, or 5 μg ml^−1^ RNA41 for 24 h. (Top) Representative Western blots of MFN1 and MFN2 with antibodies against MFN1, MFN2, and ACTB. (Bottom) Densitometric analysis of blots, *n* = 4. Download FIG S4, TIF file, 2.9 MB. Copyright © 2019 Campbell et al. 2019 Campbell et al. This content is distributed under the terms of the Creative Commons Attribution 4.0 International license.

We next assessed the effect of *TREM1* silencing on mitochondrial integrity in HIV-infected cells by determining mitofusin expression post-*TREM1* RNAi treatment. *TREM1* silencing resulted in a significant reduction in HIV-induced MFN1 and MFN2 to levels similar to those seen with uninfected cells (*P* > 0.3; Fig. 9B). When we exposed *TREM1*-silenced uninfected macrophages to HIV Tat, gp120, or RNA40, each still increased MFN1 expression, although the increase was appreciably less than in the siNS cells (Fig. S4B). In contrast, *TREM1* silencing reduced MFN2 expression in all treatments, including the vehicle treatment. Notably, this decrease was significantly enhanced post-HIV Tat treatment (mean, 87% ± 1.5% reduction; *P* < 0.0001), gp120 treatment (mean, 89% ± 0.5% reduction; *P* = 0.0001), and RNA40 treatment (mean, 85% ± 1.5% reduction; *P* = 0.0007) compared with the vehicle. These data suggest that TREM1 expression is required for the HIV-mediated upregulation of mitofusins.

## DISCUSSION

Efforts to purge the latent HIV reservoir predominantly involve reactivation of viral production from latently infected CD4^+^ T cells followed by clearance of these cells through a combination of viral and cell-mediated cytotoxicity while ART prevents subsequent rounds of infection. Although this strategy has shown promise in cell lines, it has been ineffective on primary resting CD4^+^ T cells from patients on suppressive ART and in *in vivo* studies (36–40). This approach is even less effective in macrophages, as HIV-infected macrophages are particularly resistant to HIV-mediated apoptosis and CD8^+^ T cell-mediated killing (8–10). Thus, we require an improved understanding of the precise mechanisms underlying resistance of macrophage and microglia to cytopathic effects of HIV in order to develop approaches capable of efficiently and effectively eliminating infected cells. Depleting HIV-infected macrophages may result in the decline of cytokine and chemokine production and hence suppress the excessive inflammation and aberrant immune cell recruitment that persists despite suppressive ART and which has been associated with poor prognosis and ongoing viral replication. The present study demonstrated that HIV upregulates the expression of TREM1 in macrophages and that expression of TREM1 is essential in protecting HIV-infected macrophages from HIV-induced apoptosis.

Infected macrophages display an increase in the levels of expression and recruitment of the proapoptotic protein BCL2L11 into mitochondria. Proapoptotic BH3-only proteins such as BCL2L11 bind to antiapoptotic proteins, allowing the proapoptotic multidomain proteins BAX and BAK1 to oligomerize and form channels on the mitochondrial membrane and leading to CYCS release and apoptosis. It is possible that the observed upregulation and sequestration of BCL2L11 into the mitochondria of HIV-infected macrophages are early steps in the apoptotic response to a viral infection, a response that HIV subsequently blocks downstream as we do not observe the release of CYCS from mitochondria nor an increase in apoptosis once infection is established. However, when we silenced *TREM1*, the expression levels of BCL2, BCLXL, and mitofusins all decreased, while the expression levels of BAX and BAD remained unchanged. This coincided with the release of CYCS from mitochondria and an increase in apoptosis.

Although TREM1 activity was initially described only in the context of bacterial or fungal infections, a role for TREM1 during viral infections is emerging. Early studies focused on and demonstrated that TREM1 signaling modulates pattern recognition receptor virus-associated inflammation and that TLR signaling upregulates TREM1 expression. TLR3, TLR7, TLR8, and TLR9 are all common TLRs that respond to viral PAMPs. For example, TREM1-activated murine bone marrow-derived dendritic cells display increased tumor necrosis factor (TNF) expression after treatment using CpG DNA (a TLR9 ligand) (41). Similarly, TREM1-activated PBMC display enhanced production of TNF following stimulation with either CpG DNA or the TLR3 ligand poly(I•C) (42); poly(I•C) induces transcription of *TREM1* in human primary monocytes (43). In neutrophils, TREM1 activation acts synergistically with TLR7 and TLR8 ligand-mediated activation with regard to effector mechanisms but does so antagonistically for survival (44). In the present study, we demonstrated that RNA40, a TLR8 ligand, upregulates TREM1 expression and that TREM1 is required for macrophage survival. With regard to viral infections, West Nile virus (WNV) increases *TREM1* expression in murine macrophages and dendritic cells (45) and enhances TREM1 signaling in murine livers (46). Consistent with this, exogenous activation of TREM1 increases expression levels of the interferon alpha 2 gene (*IFNA2*), *TNF*, and the interleukin-6 gene (*IL-6*) in WNV-infected murine embryonic fibroblasts, while antagonizing TREM1 leads to a reduction in their expression, suggesting that TREM1 plays a role in modulating the inflammatory response to WNV (45). Similarly, Zika virus also upregulates TREM1 signaling (47). Furthermore, the Filoviridae Marburg virus and Ebola virus both upregulate the expression and activation of TREM1 on neutrophils following internalization (20). Although Ebola virus does not replicate in these cells, it is possible that Ebola virus glycoprotein, which interacts with neutrophils, acts as a ligand for TREM1 and contributes to the cytokine storm associated with the terminal stage of Ebola virus infection (20, 48, 49). While we showed that HIV infection of macrophages increases TREM1 expression and is essential for cell survival, the role of TREM1 in the inflammatory response to HIV infection remains to be elucidated.

Cells also shed soluble TREM1 (sTREM1) from cell membranes. Although the role of sTREM1 in the context of viral pathogenesis is unknown, sTREM1 levels are increased during infection with a number of viruses, including Crimean-Congo hemorrhagic fever orthonairovirus (50), hepatitis B virus (51), WNV (45), dengue virus (52), and hepatitis C virus (51). It is possible that such increases in the levels of sTREM1 could represent a virally induced compensatory mechanism to counteract inflammatory processes. Alternatively, such increases could represent a host-induced mechanism to control tissue damage by attenuating downstream inflammatory signals. Studies are required to elucidate this function and to identify the potential of sTREM1 as a marker of disease severity in acute infections by such viruses as influenza virus and dengue virus or in chronic infections such as HIV.

Evading apoptosis by upregulation of antiapoptotic or downregulation of proapoptotic proteins is an important step in altering cell survival. Importantly, the inhibition of macrophage apoptosis may contribute to viral latency and persistence during HIV infection (10, 53). In summary, this study demonstrated an important role and a mechanism for TREM1 in prolonging survival of HIV-infected macrophages. Our report also highlights TREM1 as a therapeutic target to clear the macrophage HIV reservoir and suggests that current HIV reservoir paradigms include the targeting of TREM1 in any effort to achieve HIV eradication.

## MATERIALS AND METHODS

### Macrophages.

Venous blood was drawn from human subjects using protocols approved by the Human Research Protections Program of the University of California, San Diego, in accordance with the requirements of the Code of Federal Regulations (CFR) on the Protection of Human Subjects (45 CFR 46 and 21 CFR 50 and 56). All volunteers gave written informed consent prior to their participation. PBMC were isolated from whole blood by density gradient centrifugation over Ficoll-Paque Plus (GE Healthcare). Macrophages were prepared by incubating 6 × 10^6^ PBMC ml^−1^ in macrophage media (RPMI 1640 [Gibco] supplemented with 10% [vol/vol] heat-inactivated fetal bovine serum [FBS; Sigma]; 2 mM l-glutamine, 0.1 mg ml^−1^ streptomycin, and 100 U ml^−1^ penicillin [all Gibco]; and 10 ng ml^−1^ CSF1 (Peprotech]), after which nonadherent cells were removed by aspiration and washed with Dulbecco’s phosphate-buffered saline (Gibco). Adherent cells were further incubated in macrophage media for 10 days at 37°C and 5% CO_2_, with medium changes performed every 2 days, before use. This protocol results in a high proportion of CD14^+^ sialic acid binding Ig-like lectin 1 (SIGLEC1)^Lo^ macrophages that are permissive to HIV infection (54, 55).

### HIV.

HIV Ba-L was obtained through the NIH AIDS Reagent Program from Suzanne Gartner, Mikulas Popovic, and Robert Gallo (56, 57). Virus stocks were prepared as previously described (58). HIV infectivity levels were calculated as the 50% tissue culture infectious doses (TCID_50_) as described previously (59) and multiplicity of infection (MOI) levels confirmed using TZM-bl cells (from John C. Kappes, Xiaoyun Wu, and Tranzyme Inc.) (60). Macrophages were infected with HIV at an MOI of 0.04 unless otherwise stated.

### Chemicals.

HIV_Ba-L_ gp120 (AAA44191.1) was obtained through the NIH AIDS Reagent Program. LyoVec precomplexed RNA40 (catalog no. tlrl-lrna40) and LyoVec precomplexed RNA41 (a derivative of RNA40 in which adenosine replaced all uracil nucleotides; catalog no. tlrl-lrna41) were purchased from InvivoGen. Full-length HIV Tat (B.FR.83.HXB2; K03455 [61]) was prepared on 0.5 mmol 4(hydroxymethyl)phenoxymethyl-copolystyrene-1% divinylbenzene-resin (Applied Biosystems) using an Applied Biosystems 433A peptide synthesizer with FastMoc Chemistry as previously described (62). Peptides were deprotected and cleaved from the support using trifluoroacetic acid supplemented with 5% (vol/vol) H_2_O, 10% (vol/vol) (methylsulfanyl)benzene, and 5% (vol/vol) ethane-1,2-dithiol. Crude product was precipitated in 2-methoxy-2-methylpropane at –20°C, washed several times, filtered, and dried under vacuum. Tat was purified by reverse-phase high-performance liquid chromatography (HPLC) as previously described (29). HPLC analysis was performed as previously described (62), amino acid analysis was performed on a Beckman model 6300 amino acid analyzer, mass spectrometry was carried out using an Ettan matrix-assisted laser desorption ionization–time of flight apparatus (Amersham Biosciences), and concentrations were determined using a Beckman DU640 UV light-visible light spectrometer and an extinction coefficient of 8,480 M^−1 ^cm^−1^ (63). Staurosporine (Sigma) was used at 1 μM. Monoclonal TREM1 antibody was purchased from R&D Systems (catalog no. MAB1278; RRID:AB_2208452).

### Apoptosis assays.

Cell death was estimated using Alexa Fluor 488-tagged ANXA5 and PI staining on a FACSCalibur flow cytometer and analyzed using CellQuest Pro (both BD Biosciences). Apoptotic ssDNA damage measurements were made using an ssDNA apoptosis enzyme-linked immunosorbent assay (ELISA) kit (Millipore) as described previously (64). To assess the extent of necrotic cell death, the lactate dehydrogenase (LDH) activity of supernatants was measured using a mixture of diaphorase/NAD^+^ and 3-(4-iodophenyl)-2-(4-nitrophenyl)-5-phenyl-2H-tetrazol-3-ium chloride/sodium 2-hydroxypropanoate and percent cytotoxicity calculated according to the protocol of the manufacturer (Roche). The mitochondrial membrane potential (Δψm) gradient was measured by flow cytometry using 50 nM 1,1′,3,3,3′,3′-hexamethylindodicarbo-cyanine iodide [DiIC1(5); Molecular Probes].

### Flow cytometry.

Intracellular staining of HIV p17 was performed as previously described (65) using rabbit anti- mouse anti-gag-p17 (Abcam catalog no. ab9067; RRID:AB_306976) followed by fluorescein isothiocyanate (FITC)-conjugated goat anti-mouse IgG (Santa Cruz Biotechnology catalog no. sc-2010; RRID:AB_631735).

### siRNA transfection.

Macrophages were transfected with Thermo Fisher Silencer Select *BCL2* (identifier [ID] s1916), *RELA* (ID s11915), *TREM1* (ID s28910), or control (catalog no. 4390846) siRNA (siNS) using Lipofectamine RNAiMAX transfection reagent (Thermo Fisher) in Opti-MEM (Gibco) according to the manufacturer’s instructions. At 48 h, cells were analyzed for target gene silencing and used in experiments. Transfection efficiency was assessed with BLOCK-iT Alexa Fluor red fluorescent control (Thermo Fisher) using flow cytometry.

### Western blotting.

The following antibodies were used: anti-ACTB from Sigma (catalog no. A2228; RRID:AB_476697); anti-TREM1 from Abcam (catalog no. ab90808; RRID:AB_2050414); and anti-BAD (catalog no. 9268S; RRID:AB_823433), anti-BAX (catalog no. 5023; RRID:AB_2744530), anti-BCL2 (catalog no. 15071; RRID:AB_2744528), anti-BCL2L11 (catalog no. 2933; RRID:AB_1030947), anti-BCLXL (catalog no. 2764; RRID:AB_2228008), anti-CASP3 (catalog no. 9662; RRID:AB_331439), anti-CASP9 (catalog no. 9502; RRID:AB_2068621), anti-CYCS (catalog no. 9502; RRID:AB_2637071), anti-H3C1 (catalog no. 4499; RRID:AB_10544537), anti-MFN1 (catalog no. 14739; RRID: AB_2744531), anti-MFN2 (catalog no. 9482; RRID: AB_2716838), anti-NFKB1 (catalog no. 13586; RRID:AB_2665516), and anti-RELA (catalog no. 8242; RRID:AB_10859369) from Cell Signaling Technology.

Whole-cell lysates were prepared using 20 mM HEPES (Gibco), 150 mM NaCl (Fisher), and 1 mM EDTA (Sigma) supplemented with 1% (vol/vol) Triton X-100 (Sigma) and 1% (vol/vol) Halt protease and phosphatase inhibitor cocktail (Thermo Scientific). Cytoplasmic fractions for parallel mitochondrion analysis were prepared using a mixture containing 220 mM d-mannitol, 68 mM sucrose, 50 mM piperazine–*N*,*N*′-bis(2-ethanesulfonic acid)–KOH (pH 7.4), 50 mM KCl, 5 mM EGTA, 2 mM MgCl_2_, 1 mM dithiothreitol (all Sigma), and 1% (vol/vol) Halt protease and phosphatase inhibitor cocktail. After 30 min on ice, cell homogenates were spun at 14,000 × *g* for 15 min and supernatants harvested. For mitochondrion preparations, 1 × 10^7^ macrophages were scraped and pelleted by centrifugation at 800 × *g* for 10 min. Cells were resuspended in 1.2 ml cold mitolysis buffer (20 mM HEPES [pH 7.5], 10 mM KCl, 1.5 mM MgCl_2_, 1 mM EDTA, 1 mM EGTA, 1 mM dithiothreitol, 1 mM phenylmethanesulfonyl fluoride [all Sigma]) and incubated for 3 min on ice. Cells were homogenized with 10 strokes of a 27-gauge needle and centrifuged at 750 × *g* for 15 min at 4°C. The supernatant (cytosolic fraction) was removed, and the pellet containing mitochondria was resolved in 50 μl mitolysis buffer. Cytoplasmic and nuclear fractions were prepared for coimmunoprecipitation and EMSA using NE-PER nuclear and cytoplasmic extraction reagent according to the protocol of the manufacturer (Thermo Fisher). For coimmunoprecipitation, 50 μg anti-NFKB1 (Cell Signaling Technology catalog no. 12540; RRID:AB_2687614) was immobilized using a coupling gel and then 50-μg amounts of cell lysates were incubated with the antibody-immobilized coupling gel using a Thermo Scientific-Pierce coimmunoprecipitation kit. Lysates were resolved using a 2-[bis(2-hydroxyethyl)amino]-2-(hydroxymethyl)propane-1,3-diol-buffered 12% polyacrylamide gel and transferred to 0.2-μm-pore-size nitrocellulose membranes (Thermo Scientific), followed by detection with primary antibodies, alkaline phosphatase-tagged secondary antibodies (Invitrogen), and 0.25 mM disodium 2-chloro-5-(4-methoxyspiro {1,2-dioxetane-3,2′-(5′-chloro)tricyclo[3.3.1.13,7]decan}-4-yl)-1-phenyl phosphate supplemented with 5% (vol/vol) Nitro-Block II (both Applied Biosystems). The densities of the target bands were compared to that of the applicable reference, i.e., ACTB (for whole-cell lysates and cytoplasm fractions), COX4I1 (for mitochondrion fractions), or H3C1 (for nuclear fractions), and the data were calculated using ImageJ (NIH).

### Electrophoretic mobility shift assay.

EMSA was conducted using a LightShift chemiluminescent EMSA kit (Thermo Fisher) according to the manufacturer’s instructions using the following biotin end-labeled duplex DNA oligonucleotide: 5′-AAGTGTTTCACTCTGGATCTTATCCTTCAAAGGTCCACTTTTAAAAATTT-3′. For supershift experiments, nuclear extracts were preincubated with one of the following antibodies: anti-NFKB1 (catalog no. 13586; RRID:AB_2665516), anti-NFKB2 (catalog no. 3017; RRID:AB_10697356), anti-REL (catalog no. 67489; RRID:AB_2799726), or anti-RELA (catalog no. 8242; RRID:AB_10859369) from Cell Signaling or anti-BCL3 (catalog no. PA5-28783; RRID:AB_2546259) from Thermo Fisher. Binding reactions were resolved on a 3% to 12% NativePAGE Bis-Tris gel (Thermo Fisher) and transferred to nylon membranes (Thermo Fisher).

### Statistics.

Sample sizes are stated in the figure legends and refer to the number of independent replicates (*n*). Data are represented as dot blots with means ± 95% confidence intervals. Data were assessed for symmetry, or skewness, using Pearson’s skewness coefficient. Normalized ratiometric data were log_2_ transformed. When the level of protein expression in the reference sample was zero, we used proportion statistics to detect differences (58, 66). Comparisons between groups were performed using the paired, two-tailed Student's *t* test. In all experiments, differences were considered significant when the *P* value was less than 0.05 (*, *P* < 0.05).
