# Supplementary figures and images for: TREM-1 Protects HIV-1-Infected Macrophages from Apoptosis through Maintenance of Mitochondrial Function
Source: mBio. 2019 Nov 12;10(6):e02638-19. doi: 10.1128/mBio.02638-19 (PMC6851287; doi:10.1128/mBio.02638-19)

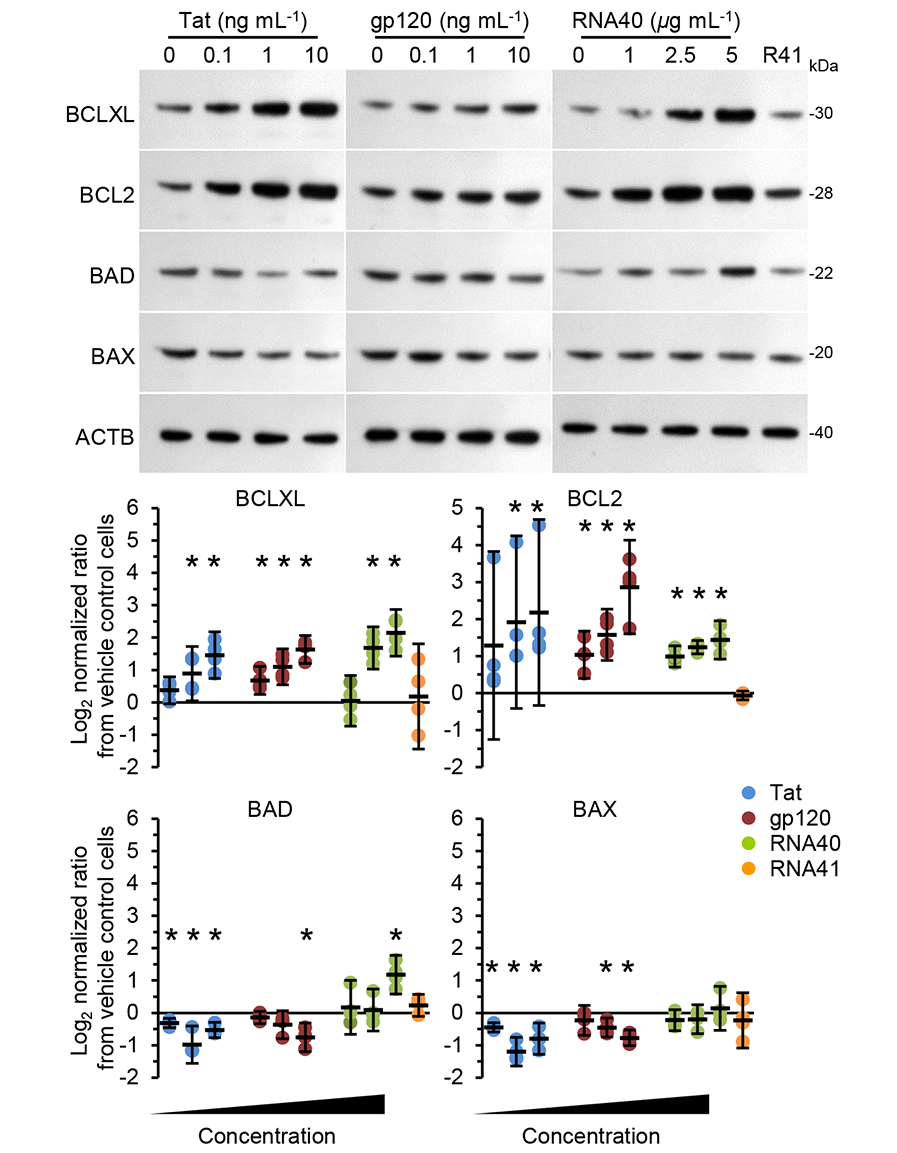

Supplement: FIG S1 [file mBio.02638-19-sf001.tif]

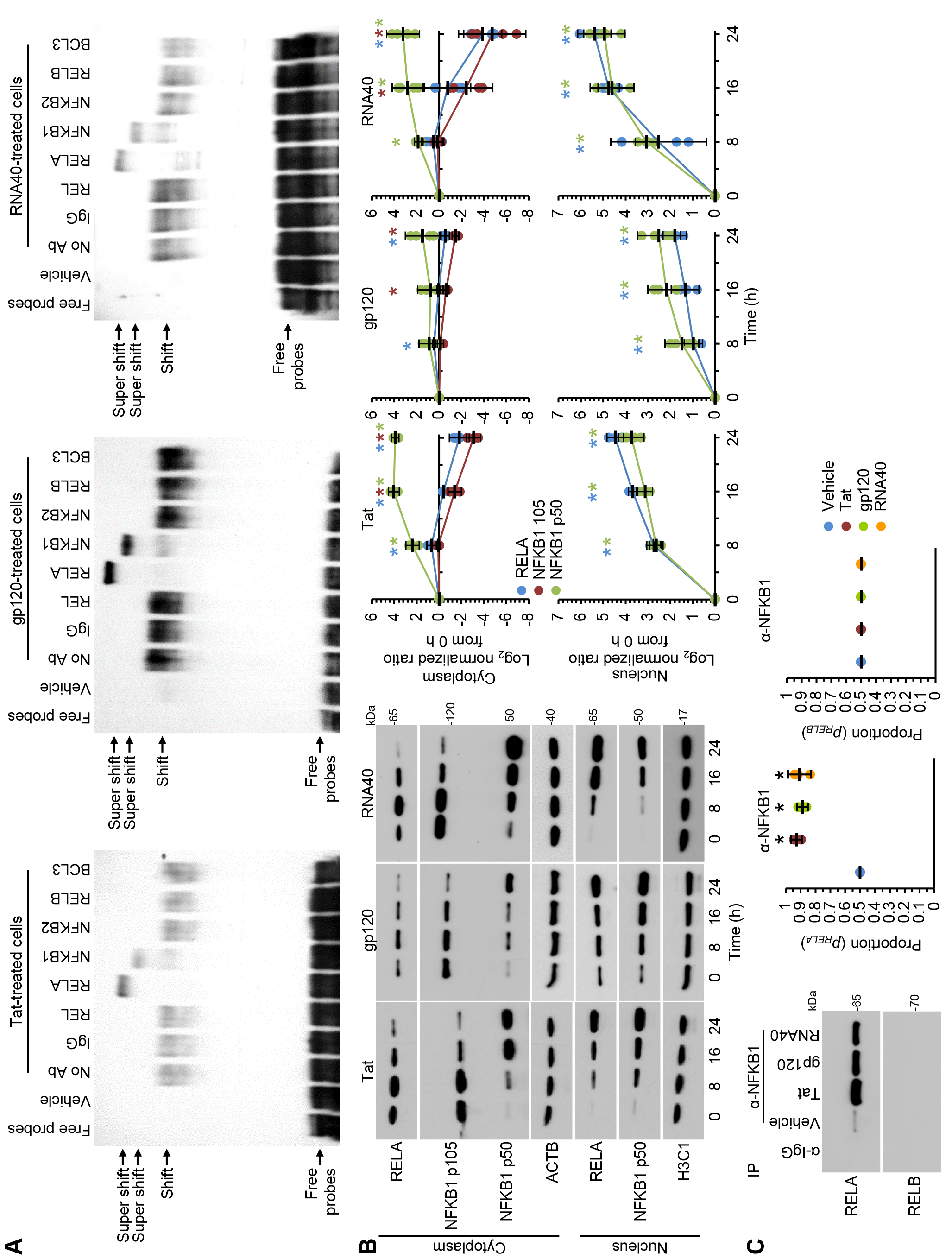

Supplement: FIG S2 [file mBio.02638-19-sf002.tif]

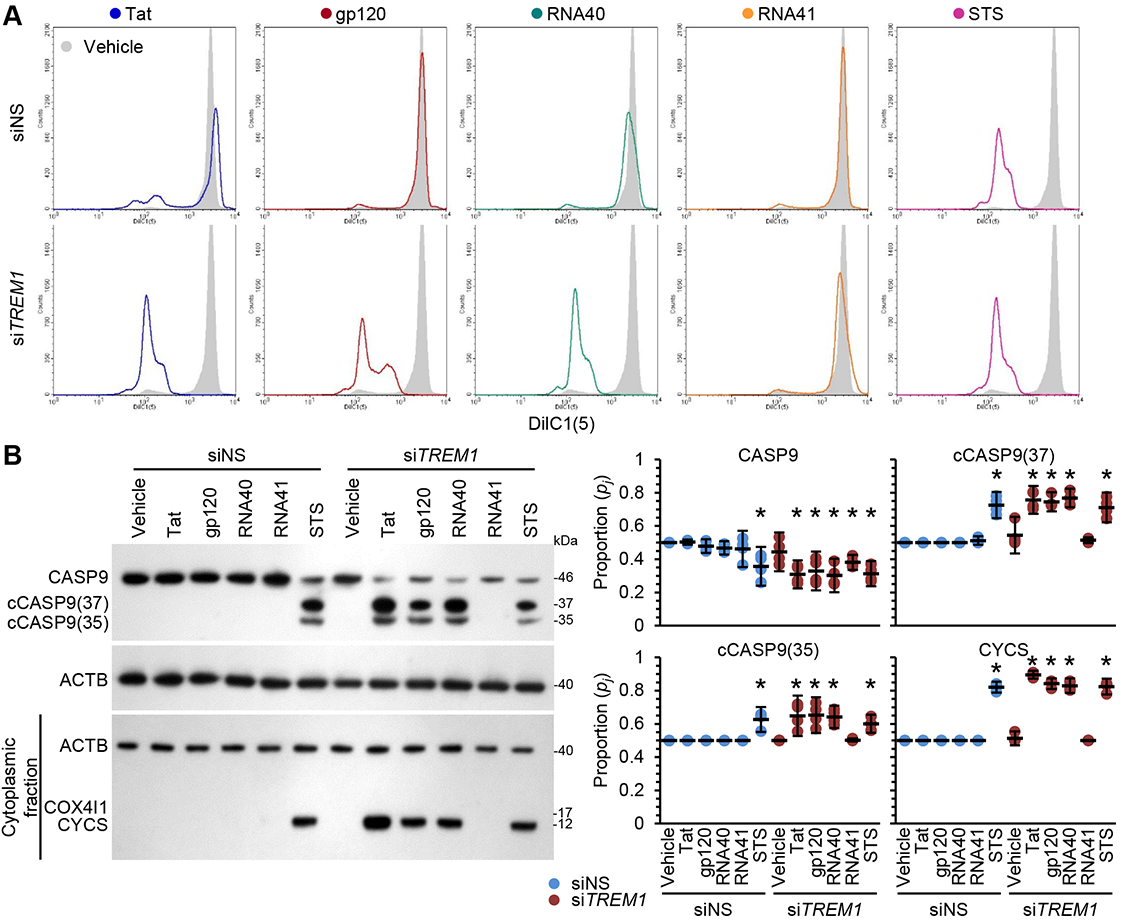

Supplement: FIG S3 [file mBio.02638-19-sf003.tif]

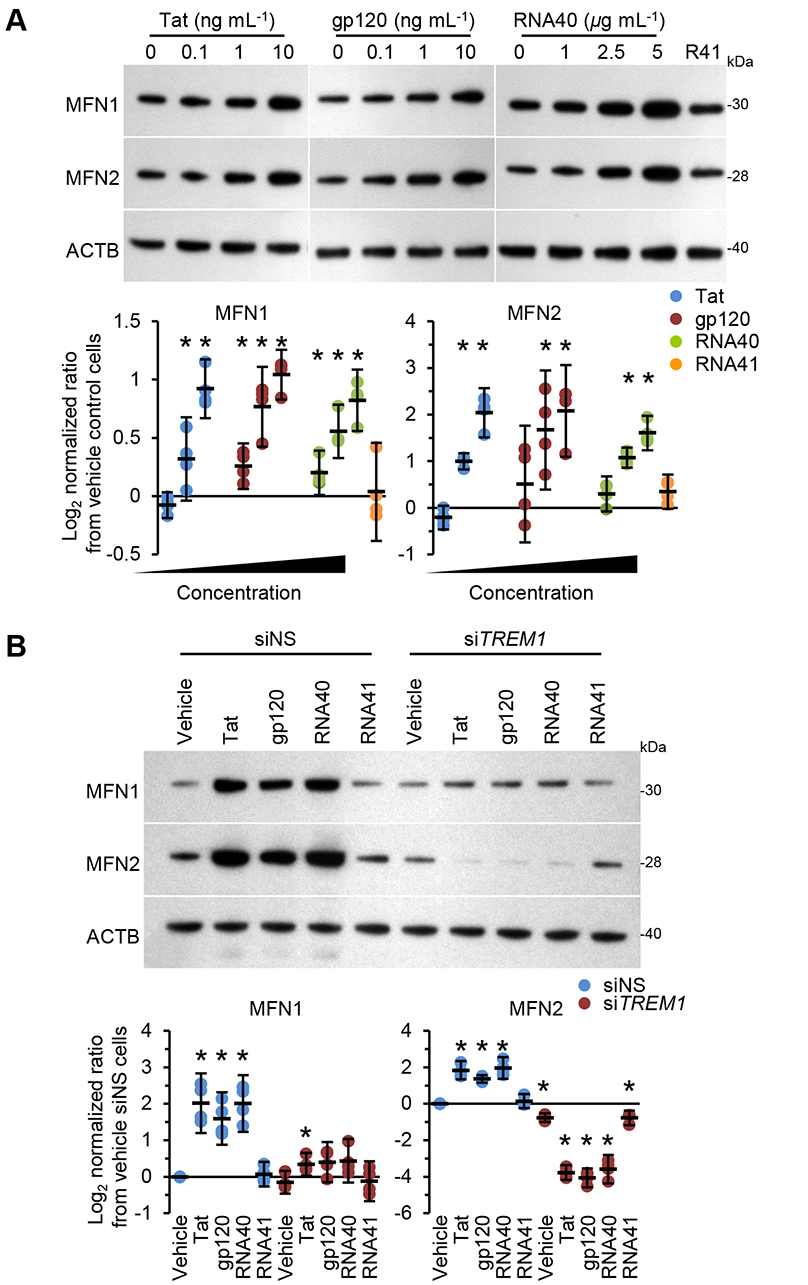

Supplement: FIG S4 [file mBio.02638-19-sf004.tif]
